# Self-Assembly of 1D/2D Hybrid Nanostructures Consisting of a Cd(II) Coordination Polymer and NiAl-Layered Double Hydroxides

**DOI:** 10.3390/polym8010005

**Published:** 2015-12-29

**Authors:** Gonzalo Abellán, Pilar Amo-Ochoa, José Luis G. Fierro, Antonio Ribera, Eugenio Coronado, Félix Zamora

**Affiliations:** 1 Instituto de Ciencia Molecular, Universidad de Valencia, Catedrático José Beltrán 2, Paterna, Valencia 46980, Spain; gonzalo.abellan@fau.de (G.A.); antonio.ribera@uv.es (A.R.); 2 Department of Chemistry and Pharmacy and Institute of Advanced Materials and Processes (ZMP), University Erlangen-Nuremberg, Henkestr. 42, Erlangen 91054 and Dr.-Mack Str. 81, Fürth 90762, Germany; 3 Departamento de Química Inorgánica and Condensed Matter Physics Center (IFIMAC), Universidad Autónoma de Madrid, Madrid 28049, Spain; pilar.amo@uam.es; 4 Instituto de Catálisis y Petroleoquímica, Cantoblanco, Madrid 28049, Spain; jlgfierro@icp.csic.es; 5 Instituto Madrileño de Estudios Avanzados en Nanociencia (IMDEA Nanociencia), Cantoblanco, Madrid 28049, Spain

**Keywords:** hybrid materials, layered double hydroxides, coordination polymers, magnetic properties, layered compounds

## Abstract

The preparation and characterization of a novel hybrid material based on the combination of a 2D-layered double hydroxide (LDH) nanosheets and a 1D-coordination polymer (1D-CP) has been achieved through a simple mixture of suspensions of both building blocks via an exfoliation/restacking approach. The hybrid material has been thoroughly characterized demonstrating that the 1D-CP moieties are intercalated as well as adsorbed on the surface of the LDH, giving rise to a layered assembly with the coexistence of the functionalities of their initial constituents. This hybrid represents the first example of the assembly of 1D/2D nanomaterials combining LDH with CP and opens the door for a plethora of different functional hybrid systems.

## 1. Introduction

Nanostructured hybrid and bio-hybrid systems are one of the main research topics in materials science for developing functional and structural advanced materials potentially useful for several technological applications [1,2]. Many different types of constituents can be combined to obtain hybrid systems using diverse strategies with the aim of enhancing their properties or creating multifunctional materials. Hybrids can be formed by combining purely inorganic materials, inorganic with organic materials, or even using biomaterials. Fabrication methods can follow two approaches, bottom-up or top-down. Typical bottom-up examples are based on sol-gel processes between silica matrices entrapping organic or biological species leading to the building of the hybrid materials, and those consisting in the direct assembly of silica-based blocks and organic molecules to introduce functionality to the inorganic counterpart [3,4].

Layered double hydroxides (LDHs) represent a wide family of anionic clays with the general formula [M^II^_1-*x*_M^III^*_x_*(OH)_2_]*^x^*^+^[A*^n^*^−^]*_x_*_/*n*_·*m*H_2_O (A*^n^*^−^ = organic molecules, oxoanions, oxometallate anions, complexes, anionic polymers, *etc*.) that present layered structure and interlayer spaces containing exchangeable anions and solvation molecules [5]. Synthetic LDHs are promising materials for a large number of potential applications, including catalyst supports, pollutant removal, pharmacy, flame retardants, magnetism and sensors [5,6,7], due to their low cost of production and high chemical versatility [6]. Moreover, their lamellar structural nature allows their exfoliation to produce suspensions containing positively charged LDH nanosheets that can be used as a kind of macromolecular building block, with a high potential as macromolecular absorbers [5,8,9]. 2D materials have attracted increasing attention in recent years due to their unique morphology and their potential use in a variety of applications ranging from electronics to gas storage or separation, catalysis, ultra-sensitive sensors, and coatings, among others [10]. The production of LDH nanosheets has offered the possibility of fabricating novel multifunctional hybrids. Indeed, we have recently reported that LDH–TaS_2_ hybrids showing the unusual coexistence of superconductivity and ferromagnetism in one material are generated by chemical design [11]. The hybrid material can be designed using a chemical building-block approach in which layers having the desired functionalities are self-assembled. Additionally, LDHs have been recently used to produce new hierarchical hybrid nanocomposites, nanocarbon-LDH materials, to take advantage of the complementary properties of both components. In this context different graphene-LDH hybrid materials have been produced [12,13,14].

On the other hand, coordination polymers (CPs) are a family of compounds that can be considered the natural extension of coordination compounds towards polymerization. They are formed by the assembly of two building blocks, metal entities and the bridging ligands. Their architectures and properties are defined by the features of these two building-blocks. This control allows the formation of structures with porosity (known as porous coordination polymers, PCPs, or metal-organic frameworks, MOFs). They show interesting properties such as catalytic activity, chirality, luminescence, electrical conductivity, magnetism, spin-transition (spin-crossover), non-linear optics (NLO), porosity or zeolitic-like behaviour [15]. CPs have been used to produce some hybrid materials using, for instance, post-synthetic reactions, or the inclusion of organic guest molecules in porous coordination polymers has led to CP-organic materials [16]. The main drawback for the use of CPs as building blocks to fabricate hybrid systems is their limited processability. We have recently developed some procedures to generate suspensions containing 1D and 2D-CPs [17,18,19,20,21,22]. In particular, a system based on Cd(II) with 6-mercaptopurine (6-MPH) has been widely studied [23], but their selective organization into 2D surfaces is still a challenge of utmost importance.

With the aim to produce a novel family of multifunctional materials combining the advantages of the physical properties and chemical design of LDHs and CPs, in this work we focused on the fabrication of a novel type of hybrid based on the combination of [Ni_0.66_Al_0.33_(OH)_2_]^0.33+^ with [Cd(6-MP^2−^)_2_]*_n_*^2*n*^^−^. Thus, we have shown that suitable mixture of suspensions containing both precursors led to the production of the first LDH/1D-CP material (**1D/2D hybrid**).

## 2. Experimental Section

### 2.1. Materials

All chemicals, NiCl_2_·6H_2_O, AlCl_3_·9H_2_O, Cd(ClO_4_)_2_·H_2_O, NaNO_3_, HNO_3_, CH_3_COOH, CH_3_COONa, C_6_H_12_N_4_ (hexamethylenetetraamine, HMT), NaOH and 6-mercaptopurine (6-MPH), were used as acquired from Sigma Aldrich, Madrid, Spain. All the experiments carried out during the synthesis and exchange of the LDH system were performed under argon atmosphere.

#### 2.1.1. Synthesis of **NiAl–LDH**

The LDH was synthesized following a homogeneous precipitation method using HMT as an ammonia releasing reagent (ARR). In a typical synthesis of NiAl-CO_3_ LDH, the chloride salts of the metals were dissolved in Milli-Q purged water, in order to reach a total metal cation concentration of 0.15 M in the final solution and keeping the stoichiometric coefficient *x* = Al/(Ni + Al) at the value of *x* = 0.33 (a 2:1 Ni/Al molar ratio). Then, an aqueous solution of HMT (three times the [Al^3+^]) was added. The resulting green mixture was placed in a stainless steel Teflon lined autoclave and heated up to 140 °C on a preheated oven. After 4 days, the autoclave was cooled on a bench to room temperature and the resulting fine powder was filtered and dried in a vacuum. The pH value of the remaining solution was found to be around 8. Finally, the green precipitate was filtered, washed thoroughly with distilled water and ethanol, and vacuum-dried at room temperature. The nitrate-exchange was carried in an excess of nitrate anion. In a typical procedure, 1 g of NiAl-CO_3_ LDH was immersed in a round-bottom flask containing 1000 mL of an aqueous solution of NaNO_3_ (1.5 M) and HNO_3_ (0.005 M). This mixture was mechanically stirred under inert atmosphere during 48 h. Afterward, the resulting green product was filtered, washed thoroughly with Milli-Q water and ethanol and dried in vacuum at room temperature.

#### 2.1.2. Exfoliation of **NiAl-NO_3_ LDH**

Exfoliation of LDH was performed by dispersing the sample finely powdered in degassed formamide at a concentration of *ca.* 1 g·L^−1^. Mixture was vigorously stirred during 15 min and sonicated (Branson 5510 ultrasonic water bath, Branson Ultrasonics, Danbury, CT, USA) three times in successive intervals of 5 min. After this treatment, emulsions were left to stand under Ar atmosphere with vigorous stirring for 48 h. The resulting transparent colloidal suspensions were centrifuged at 4500 rpm for 10 min to separate particles of non-exfoliated material.

#### 2.1.3. Preparation of the **Na_2*n*_[Cd(6-MP^2−^)_2_]*_n_***

The synthesis of the precursor [Cd(6-MP)_2_·2H_2_O]*_n_* was carried out following the conditions described by Dubler and Gyr [24]. In a typical synthesis, Cd(ClO_4_)_2_·H_2_O (2.3 mmol) was added to a solution of 6-MPH·H_2_O (4.7 mmol) in 0.1 mol·L^−1^ acetic acid/sodium acetate buffer (pH 4.6). The solution was stirred for 5 min and then stirred at 60 °C for 2 days. The transparent needles formed were filtered off, washed with water and dried under vacuum. 25 mL of a water suspension of [Cd(6-MP)_2_·2H_2_O]*_n_* (0.282 g, 0.63 mmol) was treated with NaOH (0.134 g, 3.38 mmol), and stirred at 20 °C (pH 12). [23] Then, the solution was filtered off through a pad of Celite. The clear solution obtained was ready for mixing with the exfoliated **NiAl–LDH** system.

#### 2.1.4. Synthesis of **1D/2D hybrid** Material

25 mL of a clear solution of [Cd(6-MP^2−^)_2_]*_n_*^2*n*^^−^ was added to 25 mL of solution containing the exfoliated **NiAl–LDH** system. Then, the pH of the resulting solution was adjusted to 8 by addition of HCl 0.1 mol·L^−1^. Upon standing for 12 h at 20 °C the resulting pale green material was filtered, washed with water and dried in vacuum.

### 2.2. Methods

Metallic atomic composition of bulk samples was determined by means of electron probe microanalysis (EPMA) performed in a Philips SEM-XL30 (Philips, Eindhoven, The Netherlands) equipped with an EDAX microprobe. Particles morphologies, dimensions and mapping of the chemical element distribution were studied with a TECNAI G2 F20 microscope (Philips, Eindhoven, The Netherlands). PXRD patterns were obtained by means of a Philips X’Pert diffractometer (Philips, Eindhoven, The Netherlands) using the copper radiation (Cu-K_α_ radiation, λ_α_ = 1.54178 Å). Profile was collected as step scans in the 5° < 2θ < 70° range with a spot size of 0.02°. Thermogravimetric analysis of all compounds were carried out with a Mettler Toledo TGA/SDTA 851 apparatus (Mettler-Toledo, Columbus, OH, USA) in the 25–800 °C temperature range under a 10 °C·min^−1^ scan rate and an air flow of 30 mL·min^−1^. Infrared spectra were recorded in a FT-IR Nicolet 5700 spectrometer (Thermo Scientific, Waltham, MA, USA) in the 4000–400 cm ^−1^ range using powdered samples diluted in KBr pellets. These pellets were prepared just before their use (in the case of the LDH-containing samples), in order to avoid possible contamination of the sample from an anionic exchange reaction. Raman measurements (Jobin-Yvon LabRam HR 800 Raman Microscope, Palaiseau, France) were carried out at room temperature with the 532 nm line of an Ar ion laser as an excitation source. Atomic Force Microscopy (AFM) were carried out using dynamic mode in a Nanotec Electronica system (Nanotec Electronica, Madrid, Spain) operating at room temperature in ambient air conditions. The images were processed using WSxM (Nanotec Electronica, Madrid, Spain) [25]. Commercial Olympus Si/N cantilevers (Olympus Corporation, Tokyo, Japan) were used with a nominal force constant of 0.75 N·m^−1^. The surfaces used were SiO_2_ 300 nm (IMS Company, Chagrin Falls Township, OH, USA) substrates. In order to obtain reproducible results, very flat substrates were used with precisely controlled chemical functionalities, freshly prepared just before the chemical deposition. SiO_2_ surfaces were sonicated for 15 min in acetone and 15 min in 2-propanol and then dried under an argon flow. For the morphology and roughness study a suspension of 1 mg·mL^−1^ of the material in freshly water Milli-Q was prepared. The suspension was stirred for 1 min on vortex and sonicated in an ultrasonic bath at 37 Hz and 120 W for 1 min. Upon standing for 20 min at 20 °C, 20 µL of the clear supernatant was deposited by drop-casting on a cleaned SiO_2_ substrate. The sample was left for 10 min on the SiO_2_ substrate and dried under argon flow. For the characterization of the initial exfoliated NiAl–NO_3_ LDH, a freshly diluted emulsion resulting from the delamination of samples in formamide was deposited onto a clean Si wafer by spin coating at 5000 rpm. The AFM sample preparation of **1D-CP(Cd)^−^** was done following this procedure: 1 mg of crystals of compound **1D-CP(Cd)**, neutral form, was treated with 1 mL of NaOH (0.1 mol·L^−1^). The resulting solution was centrifuged at 40,000 rpm for 10 min, and a drop of the clear solution was deposited on a cleaved mica sheet previously treated with aminopropyltriethoxysilane.

The drop was left in contact with the mica for 10 min and then washed with milli-Q water. Dynamic light scattering (DLS) measurements were recorded at 25 °C with a Zetasizer Nano ZS instrument (Malvern Instrument Ltd., Grovewood, Worcestershire, UK) on a freshly exfoliated sample as described before, during 72 h.

Photoelectron spectra (XPS) were obtained with a VG Escalab 200R spectrometer (VG Scientific, East Grinstead, UK) equipped with a hemispherical electron analyser (pass energy of 50 eV) and an MgK_α_ (hν = 1253.6 eV, 1 eV = 1.6302 × 10^−19^ J) X-ray source, powered at 120 W. The kinetic energies of photoelectrons were measured using a hemispherical electron analyser working in the constant pass energy mode. The background pressure in the analysis chamber was kept below 6 × 10^−9^ mbar during data acquisition. The XPS data signals were taken in increments of 0.1 eV with dwell times of 50 ms. Binding energies were calibrated relative to the C1s peak at 284.8 eV. High resolution spectra envelopes were obtained by curve fitting synthetic peak components using the software “XPS peak”. The raw data were used with no preliminary smoothing. Symmetric Gaussian-Lorentzian (90%G–10%L) lines were used to approximate the line shapes of the fitting components. Atomic ratios were computed from experimental intensity ratios and normalized by atomic sensitivity factors.

Magnetic susceptibility measurements were performed on polycrystalline samples with a SQUID Magnetometer MPMS-XL-5 (Quantum Design, San Diego, CA, USA). The susceptibility data were corrected by removing the diamagnetic contributions as deduced by using Pascal’s constant tables. The direct current (dc) data were collected in the range 2–300 K upon decreasing temperatures with an applied field in the range 100–5000 G. The alternating current (ac) data were collected in the range 2–10 K with an applied alternating field of 3.95 G at different frequencies in the range 1–1000 Hz. Hysteresis loops were collected between −8 and +8 T at 2 K with PPMS-9 equipment (Quantum Design, San Diego, CA, USA).

## 3. Results and Discussion

**Scheme 1:**
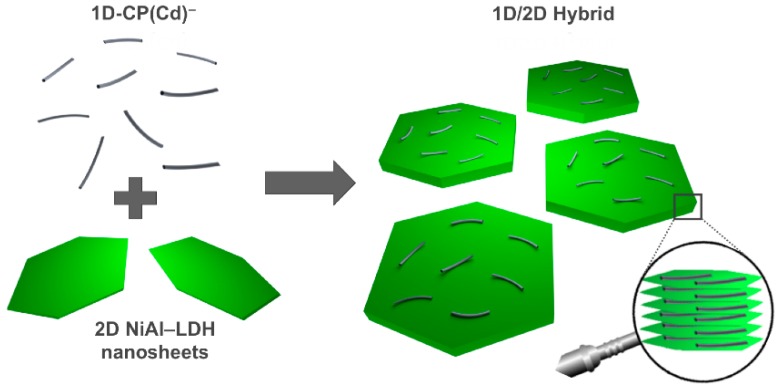
Idealized illustration of the **1D/2D hybrid** fabrication. An aqueous solution of the anionic coordination polymer [Cd(6-MP^2^^─^)_2_]*_n_*^2*n*^^−^, **1D-CP(Cd)^−^**, was mixed with a suspension of positively charged exfoliated 2D **NiAl–LDH** nanosheets in formamide, leading via electrostatic interactions to a **1D/2D hybrid** layered assembly.

The 1D/2D hybrid material (**1D/2D hybrid**) is based on the combination of an anionic [Cd(6-MP^2−^)_2_]*_n_*^2*n*^^−^ coordination polymer, **1D-CP(Cd)^−^**, and cationic **NiAl–LDH** nanosheets. It was prepared from the mixture of an exfoliated LDH suspension and fibres of the polyanion of the coordination polymer, which are produced *in situ* by treatment of the neutral polymer with an aqueous solution of NaOH (pH 11–12) [23]. This procedure, in which the nitrate-exchanged highly crystalline LDH system has previously been exfoliated in formamide, guarantees the correct incorporation of the coordination polymer to the LDH matrix and is based on electrostatic interactions between both precursors, as can be observed in Scheme 1 [5,13,26]. In addition, the exfoliation process guarantees a maximization of the positive charges within the LDH layers, resulting in the spontaneous self-assembly of both building blocks. Indeed, after the mixture of both components the presence of a pale green precipitate was clearly observed.

**Figure 1:**
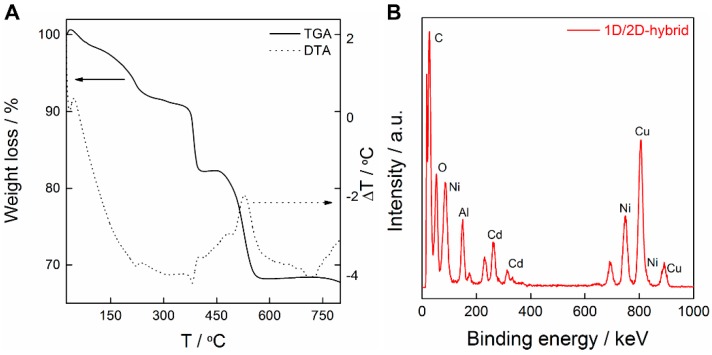
Termogravimetric (TGA/DTA) analysis (**A**) and electron probe microanalysis (**B**) of the **1D/2D hybrid**.

Figure 1A shows that the **1D/2D hybrid** display the characteristic thermogravimetric profile of **NiAl–LDH**, and also reveals the incorporation of **1D-CP(Cd)^−^** in the hybrid with an exothermic peak at *ca*. 530 °C. This mass loss represents the combustion of 14% of the mass, accounting for the decomposition of the coordination polymers as well as the partial dehydroxylation of the LDH layers, in agreement with the experimental EPMA analysis (Figure 1B, *ca*. 10 at % Cd content). In addition, the gradual weight loss of about 9% corresponds to water desorption below 240 °C. Based on the chemical analytical data obtained through EPMA (Figure 1B), the initial composition of the **NiAl–LDH** precursor (*i.e*., 2:1) is preserved after the anion exchange reaction and the subsequent exfoliation and hybridization with the Cd(II) coordination polymer.

**Figure 2:**
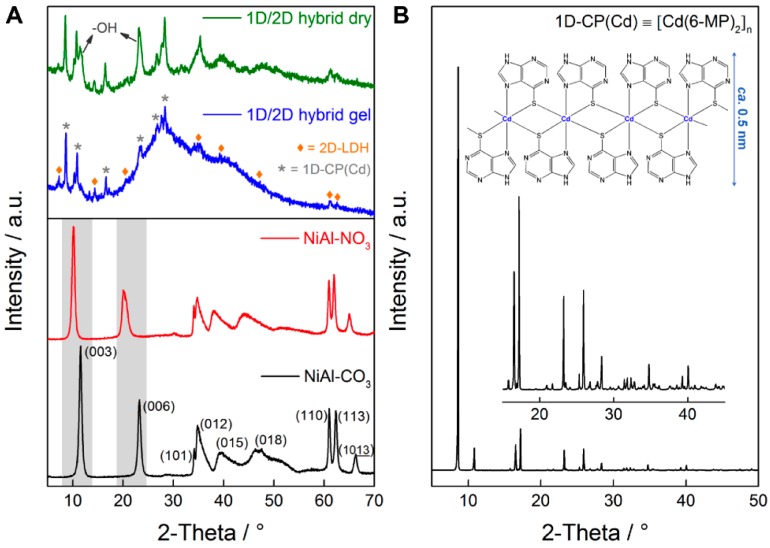
Powder X-ray diffraction patterns of the **1D/2D hybrid** and the **NiAl–LDHs** (**A**) and **1D-CP(Cd)** (**B**).

Figure 2A displays the PXRD of the LDH precursors, the initial NiAl-CO_3_ and the anion exchanged NiAl-NO_3_. The PXRD patterns show the expected profile for LDH systems with sharp peaks constantly separated at low theta values and broad and less-intense ones appearing at higher angular values. The successful anion exchange process can be clearly identified with a shift towards lower Θ values as highlighted in Figure 2A. The 1D/2D hybrid material displays a complex structure with the expected profile for LDH systems together with the corresponding peaks of the neutral coordination polymer [23,24]. Firstly we analysed the PXRD immediately after the flocculation process in the form of a wet gel-like material. By assuming the rhombohedral symmetry characteristic of LDH systems, the (00l) peaks at *ca*. 7.2°, 14.3° and 20.7° could be indexed. From the indexation of the PXRD pattern, a basal spacing (*BS*) of *ca*. 12.2 Å can be obtained. By subtracting the thickness of the brucite-like LDH layer (*ca*. 4.8 Å) a gallery height of *ca*. 7.4 Å could be calculated suggesting the presence of the **1D-CP(Cd)** in the interlamellar space (*ca*. 5 Å in diameter, *vide infra*). In addition, the PXRD pattern also exhibits the presence of the most characteristic peaks of the **1D-CP(Cd)** material (Figure 2B), indicating the absorption on the surface of the crystals.

After drying the sample during 24 h, a new series of peaks evolved, with a *BS* of *ca*. 7.6 Å indicating the incorporation of smaller anions like OH^−^ or carbonate leading to the partial restacking of the exfoliated nanosheets into a second phase. In both samples, wet gel-like material and dried, the characteristic peak of the LDH systems around 60° is present.

In order to shed light on the nature of this new phase, Raman and FT-IR spectroscopy measurements were performed. A detailed inspection of the characteristic LDH region at around 1000–1100 cm^−1^ suggest the presence of the *E*_g(R)_(OH) mode at 1062 cm^−1^, in good accordance with Kagunya *et al.* [27] (Figure S2). Moreover, FT-IR spectra revealed the absence of peaks associated with carbonate (*ca*. 1354 cm^−1^) in the 1D/2D-hybrid, in contrast to the pristine NiAl-CO_3_. These experiments point towards the formation of an OH^−^ restacked phase after drying the hybrid—an issue that will be corroborated by XPS measurements (*vide infra*). Furthermore, the typical features of **NiAl–LDH** together with the presence of the characteristic imidazole C=N and C–N vibrations at *ca*. 1620 and 1403 cm^−1^, respectively, related to the **1D-CP(Cd)** moiety can be observed (Figure S3) [23,24].

In brief, the PXRD patterns indicate that during the formation of the hybrid material, the coordination polymer is intercalated within the layers in coexistence with OH^−^, probably generating a staging compound [28,29]. Indeed, our system behaves similarly to the one described by Yang and co-workers, involving the swelling/restoration of bulk anions into LDH [30]. Similar staging phenomena arising after flocculation of exfoliated nanosheets have been described by Ma and co-workers for crown ethers, proposing two plausible models for the co-intercalation of two different anions with different molecular sizes: the Daumas–Hérold (staggered stacking in the same interlayer) and the Rüdorff (alternate different interlayers) models [31]. Finally, the **1D-CP(Cd)** moiety is also deposited over the small crystals of the **NiAl–LDH** decorating their external surface; however, this assumption would be confirmed by more specific techniques (*vide infra*). The potential of exfoliated LDHs to immobilize macromolecules was already demonstrated by Wypych and co-workers [5,32,33]. In these works they reported the effective immobilization of catalytic porphyrins on the surface of MgAl-LDHs, exhibiting an excellent behaviour in the oxidation of cyclooctene and cyclohexane using iodosylbenzene as oxidant.

**Figure 3:**
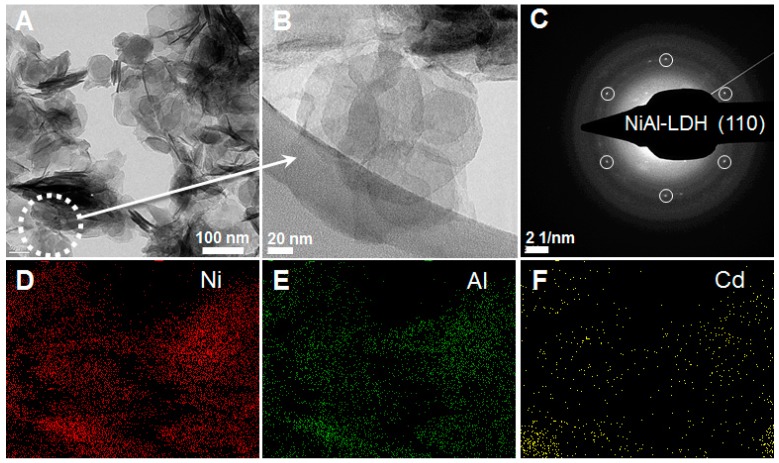
(**A**) HRTEM image of **1D/2D hybrid**; (**B**) Larger magnification of the hexagonal crystals; (**C**) SAED exhibiting a hexagonal arrangement of the diffraction peaks, as expected from the intrinsic symmetry of the LDH layers; (**D**–**F**) HRTEM-EDS elemental mapping performed over the area corresponding to (**A**) showing the homogeneous distribution of Ni, Al and Cd in the **1D/2D hybrid** material.

The morphology and crystallinity of the **1D/2D hybrid** were corroborated by HRTEM. Figure 3 shows a selection of HRTEM images highlighting the platy-like morphology associated to LDHs, with lateral dimensions in the range of 100–200 nm and thickness of less than 20 nm. Moreover, it is possible to observe the characteristic hexagonal pattern in the electron diffraction experiments, which indicates the preservation of the intrinsic hexagonal morphology of the crystals after the exfoliation and restacking process. Furthermore, we have carried out an elemental mapping with EDAX microanalysis showing a homogeneous distribution of Ni (red), Al (green) and Cd (yellow) in the crystals, discarding the presence of segregated phases and confirming that the **1D-CP(Cd)** moiety is interleaved and decorates the surface of the LDH.

**Figure 4:**
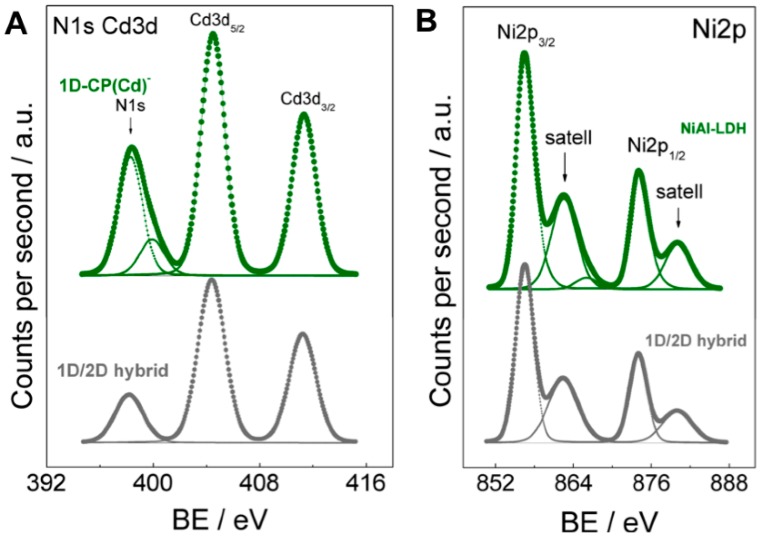
N1s and Cd3d (**A**); and Ni2p (**B**) core-level spectra of **1D-CP(Cd)**, **NiAl–LDH** and **1D/2D hybrid** samples.

The chemical state of the elements and atomic surface composition of **1D-CP(Cd)**, **NiAl–LDH** and **1D/2D hybrid** was evaluated via XPS. For each sample both the survey spectrum (Figure S3) and the high-resolution spectra of interest were recorded (Figures S4–S6). The binding energies of core levels of the elements for these samples are compiled in Table 1. The binding energy of the most intense Cd3d_5/2_ level of the Cd3d doublet of sample **1D-CP(Cd)** (404.5 eV) is similar to that of the **1D/2D hybrid** (404.8 eV) and fits with the value expected for sulphur bonded to Cd atoms (Figure 4A). Moreover, the S2p core-level spectrum of **1D-CP(Cd)** sample displays a broad peak and slight asymmetry that can be deconvoluted into two Gaussian peaks with an intensity ratio of 2:1 and spin-orbit splitting of 1.2 eV (Figure S5). The binding energy values obtained from the fitting are 161.8 and 163.0 eV, which correspond to S2p_3/2_ and S2p_1/2_ levels, respectively. These values are similar to that reported for thiol groups interacting strongly on the Cd surface [34]. The N1s peak of the **1D-CP(Cd)** sample shows two components: a major one at 398.4 eV, associated to N–C and C=N bonds of 6-MPH, and a minor one at 400.2 eV, of NH–C bonds. This latter peak disappears for the **1D/2D hybrid** sample as a consequence of the deprotonation of the hydrogen located at the N9 position of the 6-MPH ligand. Quantification surface atomic ratios were also obtained from XPS spectra. For **1D-CP(Cd)** sample an N/Cd atomic ratio of 5.97 and S/Cd atomic ratio of 1.65 are obtained, and these values are in good agreement with the expected ones (N/Cd = 8, S/Cd = 2) [23].

**Table 1:** Binding energy (eV) of core-levels of **1D-CP(Cd)**, **NiAl–LDH** and **1D/2D hybrid**.

Sample	Cd3d	S2p	N1s	Al2p	Ni2p_3/2_	C1s
1D-CP(Cd)	404.5	161.8	398.4 (76) * 400.2 (24) *	–	–	284.8
NiAl–LDH	–	–	–	74.2	856.2	288.9
1D/2D hybrid	404.8	161.8	398.3	74.5	856.9	284.8

* In parenthesis are peak percentages.

The most intense Ni2p_3/2_ peak of the Ni2p doublet of **NiAl–LDH** was around 856.2 eV and for the **1D/2D hybrid** counterpart at around 856.9 eV (Table 1 and Figure 4B). These values are substantially higher than those reported in the bibliography for pure NiO (854.5 eV), but similar to those observed for Ni_2_O_3_ (856.0 eV) and NiAl_2_O_4_ (856.3 eV) [35]. Also for this core level, satellite lines are visible, at binding energies *ca*. 6 eV higher than the main peak (at *ca*. 861.4 eV). These satellite lines are the fingerprints of Ni^2+^ ions in an environment of oxide (O^2−^) ions. Electron transfer from Ni to Al in neighbouring positions in the structure could cause the shift to a higher binding energy than that of free NiO [35]. Moreover, the **NiAl–LDH** (NiAl–CO_3_ LDH) sample shows a C1s component at high binding energy (288.9 eV) (Table 1), which is due to carbonates present in the LDH structure. Interestingly, this carbonate component in C1s line is absent in the **1D/2D hybrid** sample, in good accordance with FT-IR and Raman spectra. Additionally, **1D/2D hybrid** shows the C1s peak at 284.8 eV corresponding to the aromatic carbons of the mercaptopurine ring which is also observable in the **1D-CP(Cd)** sample (Figure S4–S6). Quantification demonstrated that the Ni/Al atomic ratio in both **NiAl–LDH** (1.29) and the **1D/2D hybrid** (1.24) remains essentially unchanged. The quantification of the Ni/Cd atomic ratio yields values of 7.61.

**Figure 5:**
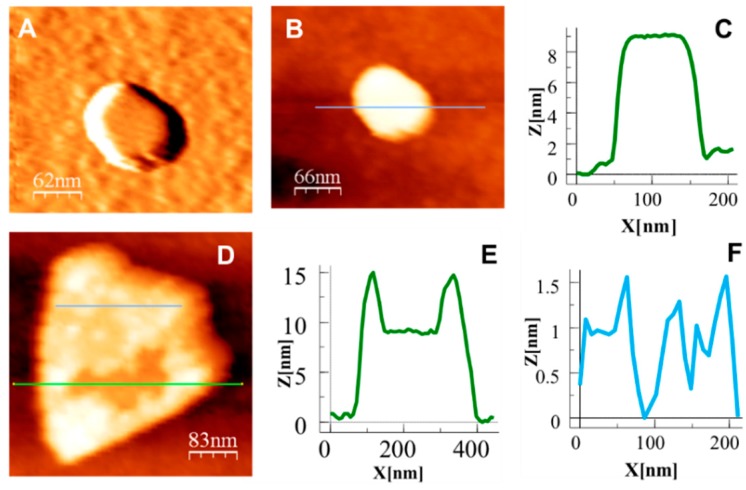
AFM analysis of an aqueous suspension of **NiAl–LDH** material drop-casted on SiO_2_ substrate. (**A**) Topographic and (**B**) derivate images showing its characteristic structural features; and (**C**) height profile of the nanosheet; (**D**) AFM topography image of the **1D/2D hybrid** nanomaterial and (**E**,**F**) its height profile analysis.

A direct evidence of the assembly between the coordination polymer and the LDH nanosheets could be provided by atomic force microscopy (AFM). Figure 5 displays the most representative AFM images of **NiAl–LDH** (NiAl-NO_3_ LDH) and **1D/2D hybrid** nanomaterials deposited by drop casting on SiO_2_ substrates (additional experiments can be found in the Supplementary Materials, Figures S9–S14). We employed the same preparation procedure using Milli-Q water as solvent, in order to avoid the presence of contaminants arising from high-boiling-point solvents like formamide on the surface of the crystals. It is worth noting that the characterization of the **NiAl–LDH** exfoliated in formamide provides an average thickness of *ca*. 1 nm—corresponding to one monolayer [5]—and lateral dimensions of *ca*. 200 nm, as determined by DLS measurements (Figures S8 and S9) [36].

The inspection of the substrates corroborates the presence of objects with homogeneity in sizes and shapes. The images confirm the presence of very thin crystals as was also observed by HRTEM. In the most particles observed it is possible to distinguish the characteristic *ca*. 120° angles reminiscent of the hexagonal shape of the original LDH particles, but the geometry of the hybrid material undergoes a significant change. The lateral dimensions and the thickness (apparent height profiles in Figure 5) of the initial **NiAl–LDH** are almost unaltered upon hybrid formation. However, the flat surface of these original crystals contrasts with the roughness observed in the surface of the **1D/2D hybrid** due to the presence of coordination polymer decorating that surface.

**Figure 6:**
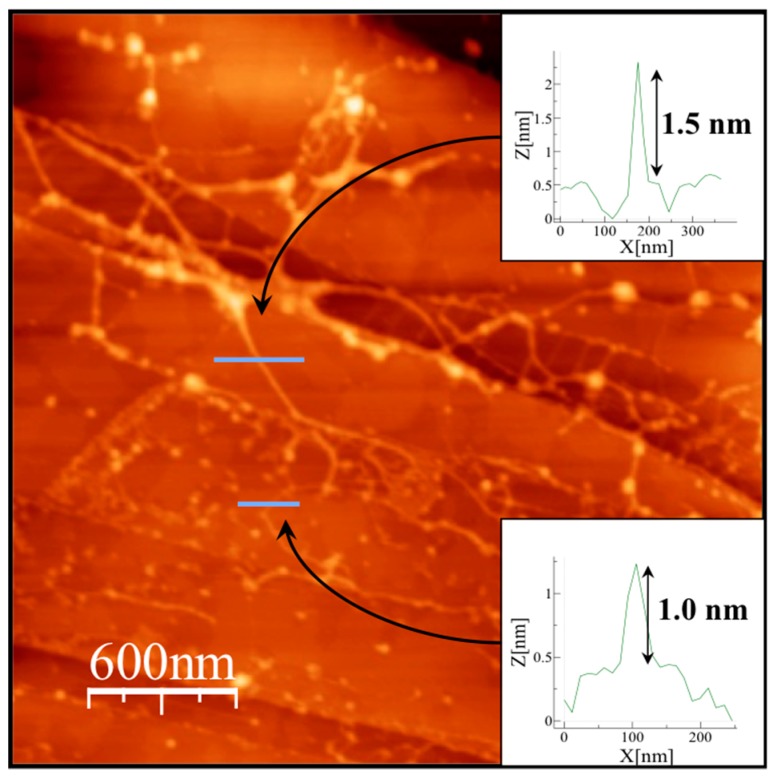
Topographic AFM image of an aqueous solution of **1D-CP(Cd)^−^** material drop-casted on a mica substrate. The insets show the height profiles highlighted on the image, indicative of the presence of **1D-CP(Cd)^−^** consisting of 2–3 chains.

We were able to measure the size of the roughness detected in some particles, compare it to the size of the coordination polymer units and observe that the diameter of the coordination polymer is (*ca*. 5 Å based on X-ray data [23]) very similar to the roughness observed by AFM (Figure 6 and Figure S14). Indeed, AFM measurements of an aqueous solution of **1D-CP(Cd)^−^** material deposited on different substrates shows the formation of 1D polymer chains consisting of aggregates of 1–3 chains (Figure 6). Therefore, we can conclude that the **1D-CP(Cd)** moiety self-assembled to the **NiAl–LDH** crystals, absorbed on their surface.

**Figure 7:**
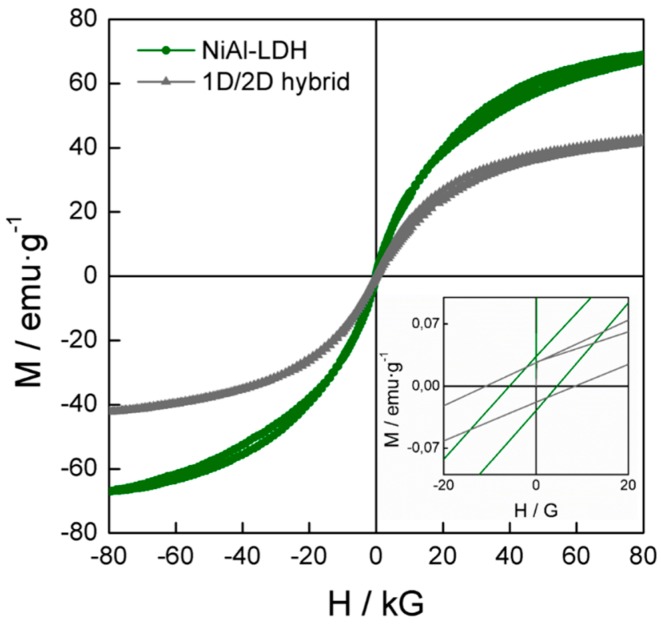
Hysteresis cycle at 2 K of the pristine **NiAl–LDH** and the **1D/2D hybrid**. The inset represents the low field region. The plot shows the dramatic magnetization decrease after the hybridization of the ferromagnetic **NiAl–LDH** with the diamagnetic **1D-CP(Cd)^−^**.

Finally, a magnetic study of the **1D/2D hybrid** was carried out (Figure 7) with the objective of checking the variations in their magnetic properties respect to the pristine **NiAl–LDH**. Both the pristine **NiAl–LDH** and the **1D/2D hybrid** material exhibited a typical low-temperature ferromagnetic behaviour characteristic of LDH systems with a temperature for the onset of the spontaneous magnetization of *ca*. 4.4 K, which reflects the fact that the intrinsic magnetic nature of the LDH layers remains intact [37,38,39].

Remarkably, the magnetization *vs.* field plot revealed a dramatic decrease in the saturation magnetization of *ca*. 38%, from 69 emu·g^−1^ for the pristine **NiAl–LDH** to 43 emu·g^−1^ for the final **1D/2D hybrid**, as well as slight variations in the coercive field. These modifications in the hysteretic behaviour can be ascribed to the contribution of the diamagnetic coordination polymer, which is intercalated as well as decorating the surface of the ferromagnetic crystals. This result opens the door for the development of related 1D/2D hybrid magnetic materials with the incorporation of magnetically active 1D coordination polymers, or even switchable molecules that will allow for controlling the properties of the LDHs platelets [40,41].

## 4. Conclusions

A hybrid material consisting of **1D-CP(Cd)** coordination polymer interleaved as well as decorating **2D-NiAl–LDH** has been achieved by means of electrostatic self-assembly through an exfoliation/restacking approach. We have characterized the obtained **1D/2D hybrid** using PXRD, TG, FT-IR, Raman, XPS, HRTEM, AFM and magnetic measurements, demonstrating that the 1D-CP are partially intercalated as well as attached to the surface of nanometric platelets of LDH of 100–200 nm in lateral dimensions, and their intrinsic properties are maintained. The hierarchical structure obtained is a good example of how, with appropriate selection of building blocks, it is possible to engineer the assembly of different nanomaterials while keeping their initial functionalities intact. To the best of our knowledge, this is the first example of a 1D/2D LDH-based material. This strategy could be easily extended to other 1D coordination polymers with interesting properties such as photoluminescence, (electro)catalytic properties, magnetic properties or even electrical conductivity.

## Supplementary Materials

Supplementary materials are available online at www.mdpi.com/2073-4360/8/1/5/s1.

## References

[1] Sanchez C., Shea K.J., Kitagawa S. (2011). Recent progress in hybrid materials science. Chem. Soc. Rev..

[2] Gómez-Romero P., Sanchez C. (2006). Functional Hybrid Materials.

[3] Ruiz-Hitzky E., Aranda P., Darder M., Ogawa M. (2011). Hybrid and biohybrid silicate based materials: Molecular *vs.* block-assembling bottom-up processes. Chem. Soc. Rev..

[4] Abellán G., Martí-Gastaldo C., Ribera A., Coronado E. (2015). Acc. Chem. Res..

[5] Wang Q., O’Hare D. (2012). Recent advances in the synthesis and application of layered double hydroxide (LDH) nanosheets. Chem. Rev..

[6] Duan X., Evans D. (2006). Layered Double Hydroxides.

[7] Rives V. (2001). Layered Double Hydroxides: Present and Future.

[8] Ma R.Z., Sasaki T. (2010). Nanosheets of oxides and hydroxides: Ultimate 2D charge-bearing functional crystallites. Adv. Mater..

[9] Abellan G., Coronado E., Marti-Gastaldo C., Pinilla-Cienfuegos E., Ribera A. (2010). Hexagonal nanosheets from the exfoliation of Ni^2+^–Fe^3+^ LDHs: A route towards layered multifunctional materials. J. Mater. Chem..

[10] Mas-Balleste R., Gomez-Navarro C., Gomez-Herrero J., Zamora F. (2011). 2D materials: To graphene and beyond. Nanoscale.

[11] Coronado E., Marti-Gastaldo C., Navarro-Moratalla E., Ribera A., Blundell S.J., Baker P.J. (2010). Coexistence of superconductivity and magnetism by chemical design. Nat. Chem..

[12] Li H.J., Zhu G., Liu Z.H., Yang Z.P., Wang Z.L. (2010). Fabrication of a hybrid graphene/layered double hydroxide material. Carbon.

[13] Latorre-Sanchez M., Atienzar P., Abellan G., Puche M., Fornes V., Ribera A., Garcia H. (2012). The synthesis of a hybrid graphene-nickel/manganese mixed oxide and its performance in lithium-ion batteries. Carbon.

[14] Zhao M.Q., Zhang Q., Huang J.Q., Wei F. (2012). Hierarchical nanocomposites derived from nanocarbons and layered double hydroxides—Properties, synthesis, and applications. Adv. Funct. Mater..

[15] Batten S.R., Neville S.M., Turner D. (2009). Coordination Polymers: Design, Analysis and Applications.

[16] Foo M.L., Matsuda R., Kitagawa S. (2014). Functional hybrid porous coordination polymers. Chem. Mater..

[17] Garcia-Couceiro U., Olea D., Castillo O., Luque A., Roman P., de Pablo P.J., Gomez-Herrero J., Zamora F. (2005). Scanning probe microscopy characterization of single chains based on a one-dimensional oxalato-bridged manganese(II) complex with 4-aminotriazole. Inorg. Chem..

[18] Olea D., Alexandre S.S., Amo-Ochoa P., Guijarro A., de Jesus F., Soler J.M., de Pablo P.J., Zamora F., Gomez-Herrero J. (2005). From coordination polymer macrocrystals to nanometric individual chains. Adv. Mater..

[19] Olea D., Garcia-Couceiro U., Castillo O., Gomez-Herrero J., Zamora F. (2007). Nanoprocessability of a one-dimensional oxalato-bridged cobalt(II) complex with 1,2,4-triazole. Inorg. Chim. Acta.

[20] Olea D., Gonzalez-Prieto R., Priego J.L., Barral M.C., de Pablo P.J., Torres M.R., Gomez-Herrero J., Jimenez-Aparicio R., Zamora F. (2007). Mmx polymer chains on surfaces. Chem. Commun..

[21] Amo-Ochoa P., Welte L., González-Prieto R., Sanz Miguel P.J., Gómez-García C.J., Mateo-Martí E., Delgado S., Gómez-Herrero J., Zamora F. (2010). Single layers of a multifunctional laminar Cu(I,II) coordination polymer. Chem. Commun..

[22] Gallego A., Hermosa C., Castillo O., Berlanga I., Gomez-Garcia C.J., Mateo-Marti E., Martinez J.I., Flores F., Gomez-Navarro C., Gomez-Herrero J. (2013). Solvent-induced delamination of a multifunctional two dimensional coordination polymer. Adv. Mater..

[23] Amo-Ochoa P., Rodriguez-Tapiador M.I., Castillo O., Olea D., Guijarro A., Alexandre S.S., Gomez-Herrero J., Zamora F. (2006). Assembling of dimeric entities of Cd(II) with 6-mercaptopurine to afford one-dimensional coordination polymers: Synthesis and scanning probe microscopy characterization. Inorg. Chem..

[24] Dubler E., Gyr E. (1988). New metal-complexes of the antitumor drug 6-mercaptopurine—Syntheses and X-ray structural characterizations of dichloro(6-mercaptopurinium)copper(I), dichlorotetrakis(6-mercaptopurine)cadmium(II), and bis(6-mercaptopurinato)cadmium(II) dihydrate. Inorg. Chem..

[25] Horcas I., Fernandez R., Gomez-Rodriguez J.M., Colchero J., Gomez-Herrero J., Baro A.M. (2007). Wsxm: A software for scanning probe microscopy and a tool for nanotechnology. Rev. Sci. Instrum..

[26] Wang L., Wang D., Dong X.Y., Zhang Z.J., Pei X.F., Chen X.J., Chen B.A., Jin J.A. (2011). Layered assembly of graphene oxide and co-al layered double hydroxide nanosheets as electrode materials for supercapacitors. Chem. Commun..

[27] Kagunya W., Baddour-Hadjean R., Kooli F., Jones W. (1998). Vibrational modes in layered double hydroxides and their calcined derivatives. Chem. Phys..

[28] Williams G.R., O’Hare D. (2005). Factors influencing staging during anion-exchange intercalation into [LiAl_2_(OH)_6_]X·*m*H_2_O (X = Cl^−^, Br^−^, NO3^−^). Chem. Mater..

[29] Ijdo W.L., Pinnavaia T.J. (1998). Staging of organic and inorganic gallery cations in layered silicate heterostructures. J. Solid State Chem..

[30] Huang G.L., Ma S.L., Zhao X.H., Yang X.J., Ooi K. (2010). Intercalation of bulk guest into ldh via osmotic swelling/restoration reaction: Control of the arrangements of thiacalix[4]arene anion intercalates. Chem. Mater..

[31] Ma S.L., Wang J., Du L., Fan C.H., Sun Y.H., Sun G.B., Yang X.J. (2013). Co-assembly of LDH nanosheets with crown ethers: Structural transformation and water-adsorption behavior. Eur. J. Inorg. Chem..

[32] Wypych F., Bubniak G.A., Halma M., Nakagaki S. (2003). Exfoliation and immobilization of anionic iron porphyrin in layered double hydroxides. J. Colloid Interface Sci..

[33] Nakagaki S., Halma M., Bail A., Arizaga G.G.C., Wypych F. (2005). First insight into catalytic activity of anionic iron porphyrins immobilized on exfoliated layered double hydroxides. J. Colloid Interface Sci..

[34] Colvin V.L., Goldstein A.N., Alivisatos A.P. (1992). Semiconductor nanocrystals covalently bound to metal-surfaces with self-assembled monolayers. J. Am. Chem. Soc..

[35] Profeti L.P.R., Dias J.A.C., Assaf J.M., Assaf E.M. (2009). Hydrogen production by steam reforming of ethanol over Ni-based catalysts promoted with noble metals. J. Power Sources.

[36] Coronado E., Marti-Gastaldo C., Navarro-Moratalla E., Ribera A., Tatay S. (2013). Illustrating the processability of magnetic layered double hydroxides: Layer-by-layer assembly of magnetic ultrathin films. Inorg. Chem..

[37] Coronado E., Galan-Mascaros J.R., Marti-Gastaldo C., Ribera A., Palacios E., Castro M., Burriel R. (2008). Spontaneous magnetization in Ni-Al and Ni-Fe layered double hydroxides. Inorg. Chem..

[38] Abellan G., Busolo F., Coronado E., Marti-Gastaldo C., Ribera A. (2012). Hybrid magnetic multilayers by intercalation of Cu(II) phthalocyanine in LDH hosts. J. Phys. Chem. C.

[39] Abellan G., Carrasco J.A., Coronado E. (2013). Room temperature magnetism in layered double hydroxides due to magnetic nanoparticles. Inorg. Chem..

[40] Abellan G., Coronado E., Marti-Gastaldo C., Ribera A., Jorda J.L., Garcia H. (2014). Photo-switching in a hybrid material made of magnetic layered double hydroxides intercalated with azobenzene molecules. Adv. Mater..

[41] Abellan G., Jorda J.L., Atienzar P., Varela M., Jaafar M., Gomez-Herrero J., Zamora F., Ribera A., Garcia H., Coronado E. (2015). Stimuli-responsive hybrid materials: Breathing in magnetic layered double hydroxides induced by a thermoresponsive molecule. Chem. Sci..

