# Supplementary material for: Self-Assembly of 1D/2D Hybrid Nanostructures Consisting of a Cd(II) Coordination Polymer and NiAl-Layered Double Hydroxides
Source: Polymers (Basel). 2015 Dec 29;8(1):5. doi: 10.3390/polym8010005 (PMC6432570; doi:10.3390/polym8010005)
Supplement: Supplementary file 1 [file polymers-08-00005-s001.pdf]

Supplementary Materials: Self-Assembly of 1D/2D Hybrid Nanostructures Consisting of Cd(II) Coordination Polymer and NiAl–Layered Double Hydroxides

Gonzalo Abellán, Pilar Amo-Ochoa, José Luis G. Fierro, Antonio Ribera, Eugenio Coronado and Félix Zamora

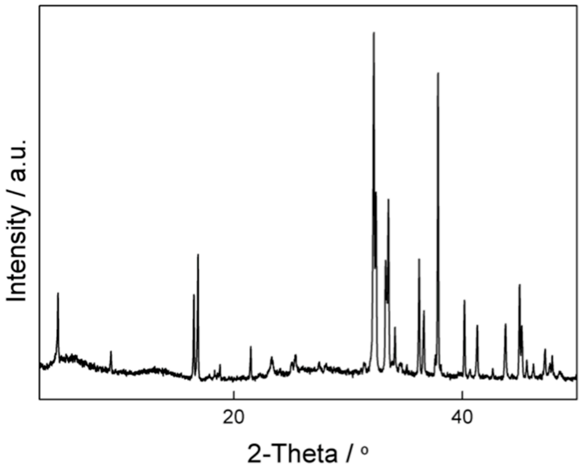

Figure S1. X-ray powder diffraction pattern of **$Na_{2n}[Cd(6\text{-}MP^{2-})_2]_n$**.

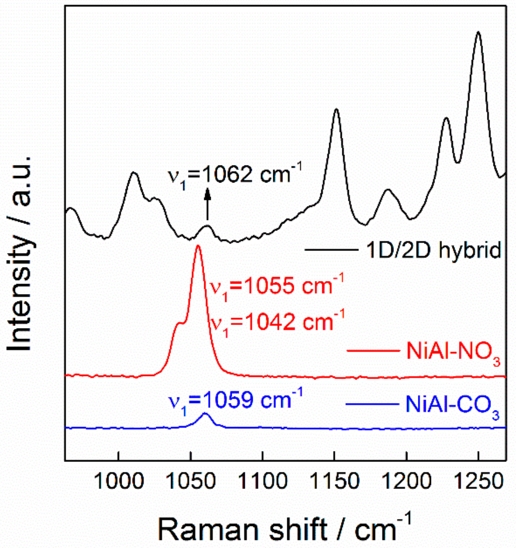

Figure S2. Raman spectra of the **$NiAl\text{-}CO_3$**, the **$NiAl\text{-}NO_3$** and the **1D/2D-hybrid**.

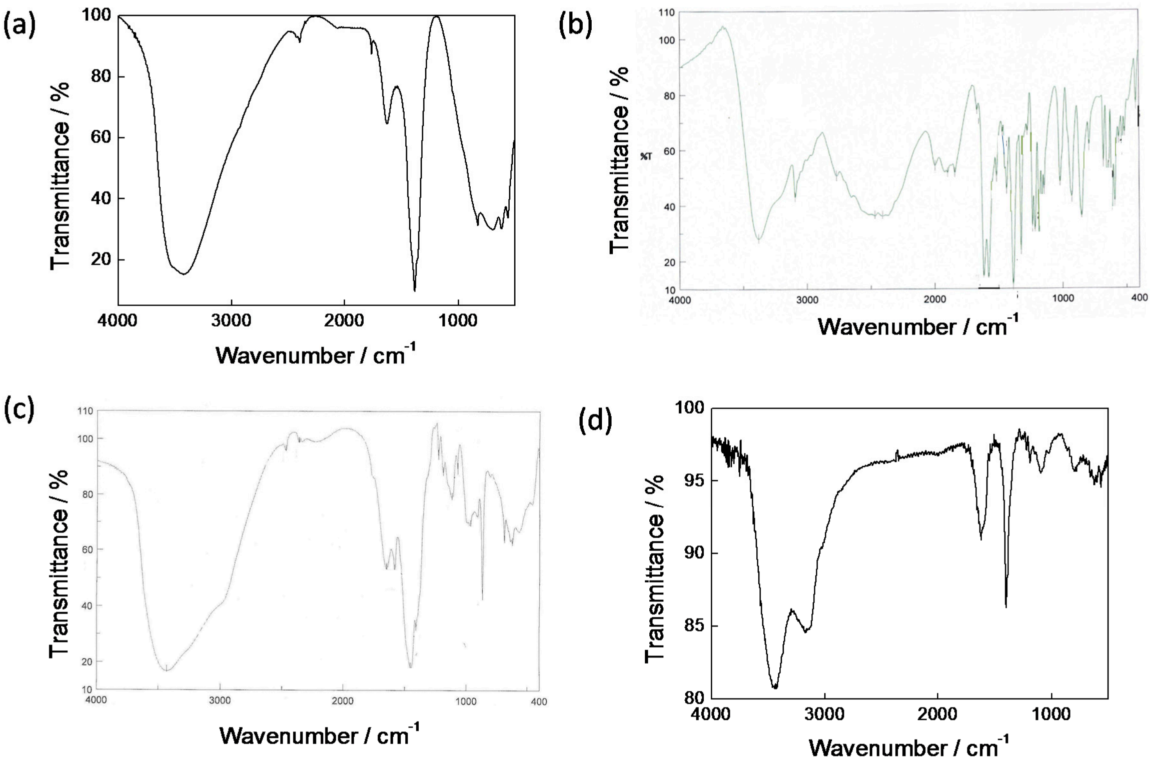

Figure S3. FTIR spectra of (**a**) **NiAl-NO$_3$ LDH**,[1] (**b**) [Cd(6-MP$^-$)$_2$.2H$_2$O]$_n$ (6-MPH= 6-mercaptopurine) **1D-CP(Cd)**; (**c**) **Na$_{2n}$[Cd(6-MP^{2-})$_2$]$_n$** and (**d**) **1D/2D hybrid** materials.

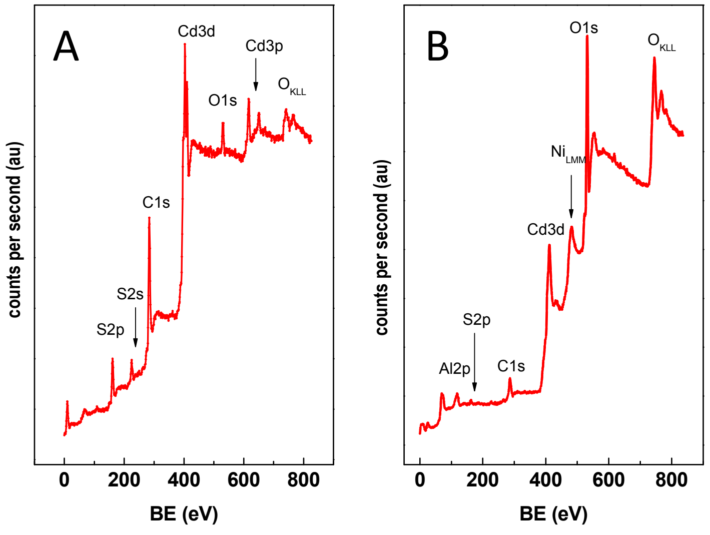

Figure S4. XPS survey spectra of [Cd(6-MP$^-$)$_2$·2H$_2$O]$_n$ and **1D/2D hybrid material.**

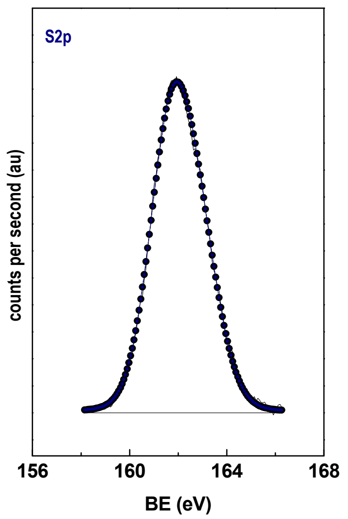

Figure S5. S2p core-level spectra of [Cd(6-MP⁻)$_2$·2H_2O]$_n$.

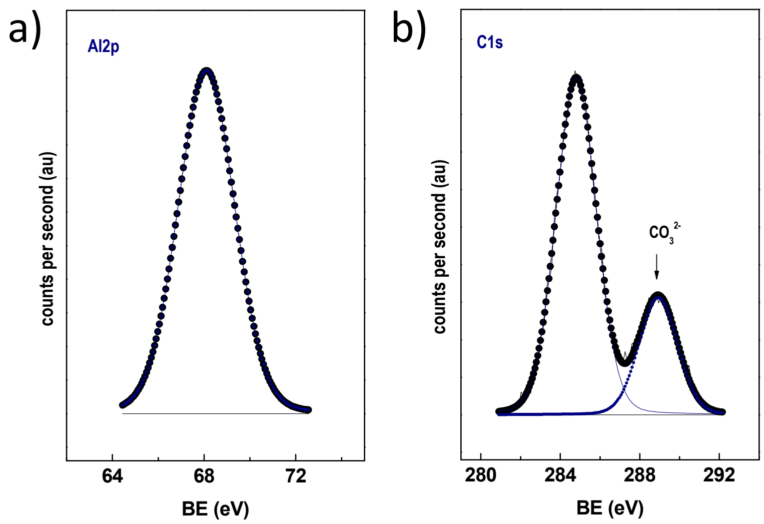

Figure S6. Al2p (**a**) and C1s (**b**) core-level spectra of **NiAl-CO_3 LDH**.

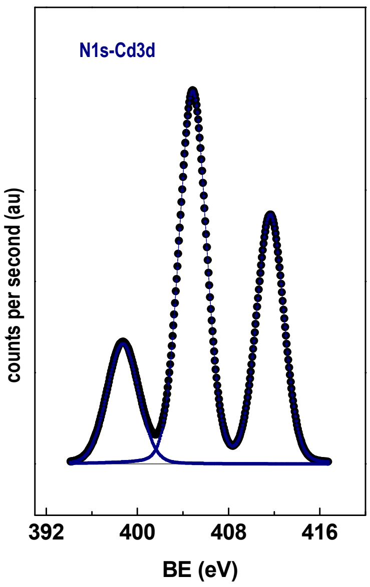

Figure S7. Ni2p core-level spectra of 1D/2D hybrid material.

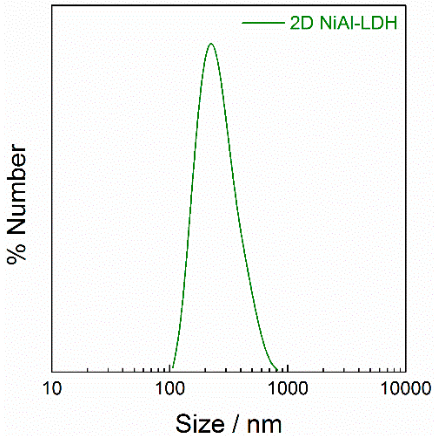

Figure S8. Dynamic light scattering (DLS) size distribution of the exfoliated NiAl-NO_3 LDH in formamide measured after 72 h, highlighting the stability of the samples [36].

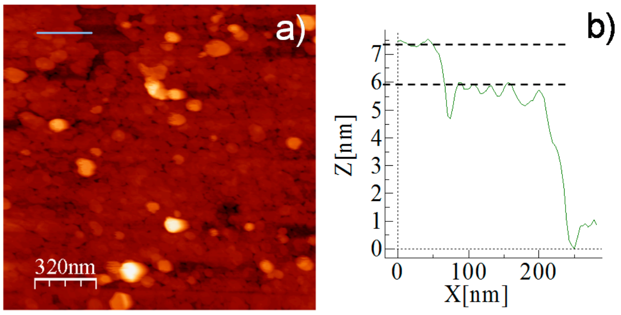

Figure S9. (**a**) Topographic AFM image on SiO_2 of the NiAl-NO_3 LDH exfoliated in formamide; and (**b**) its height profile showing a typical step of *ca*. 1 nm.

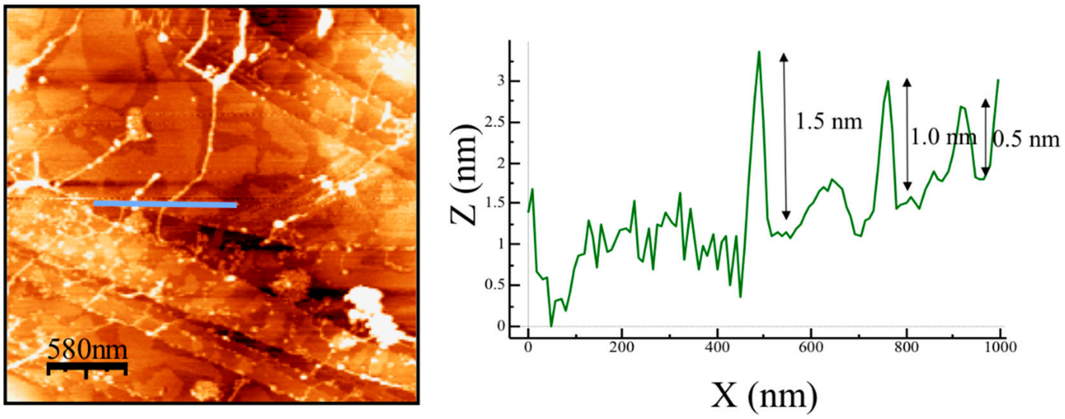

Figure S10. Topography AFM image of $[Cd(6\text{-}MP^{-})_2{\cdot}2H_2O]_n$ (**left**) and its height profile (**right**) showing from a single chain of 0.5 nm to bundles compose of 2 and 3 chains.

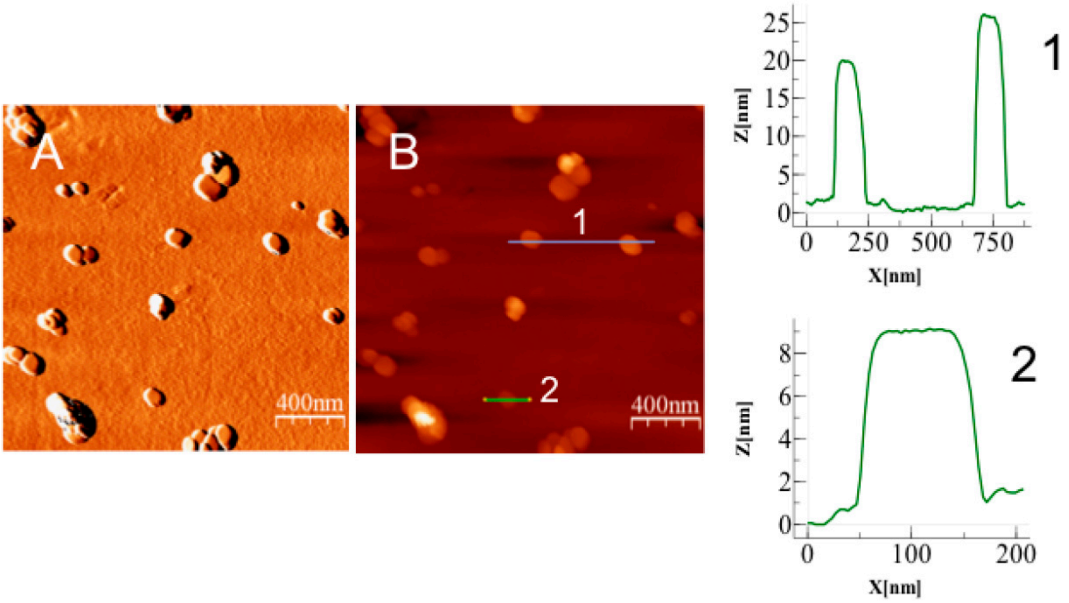

Figure S11. AFM topography image of **NiAl–LDH** material drop-casted on SiO_2 substrate. (**A**) Topographic and (**B**) derivate images with two typical height profiles (**1**,**2**).

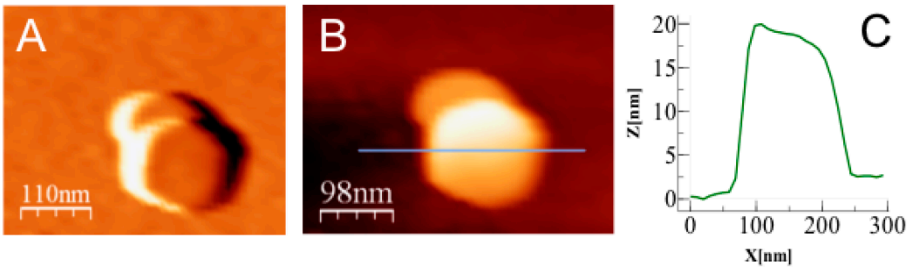

Figure S12. AFM topography image of **NiAl–LDH** material drop-casted on SiO_2 substrate. (**A**) Topographic and (**B**) derivate images and (**C**) its height profile showing the planar surface of the crystal.

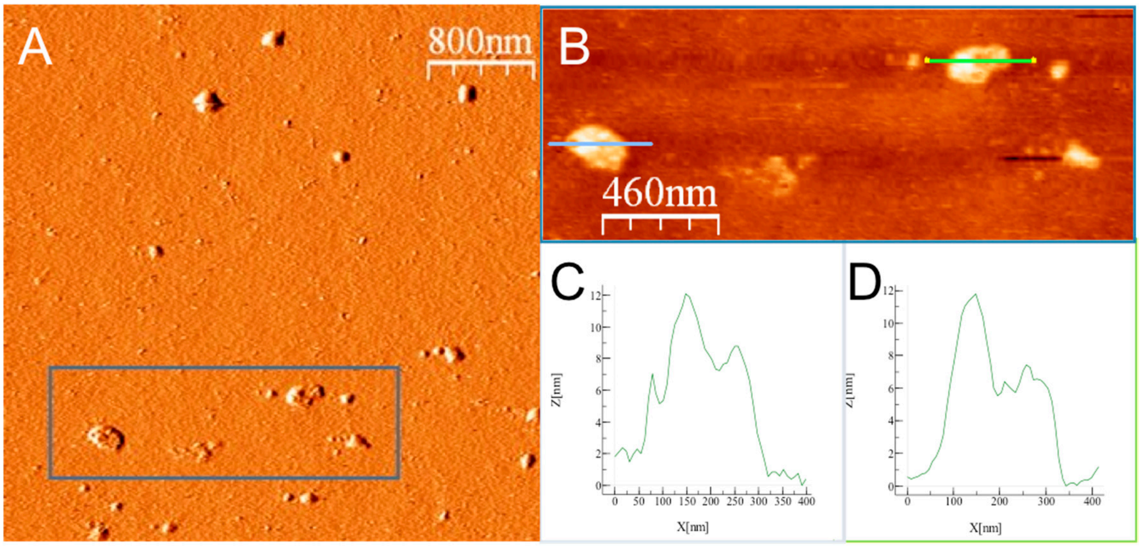

Figure S13. Large area topography AFM image of **1D/2D hybrid** material drop-casted on SiO_2 substrate (**A**); and a zoomed image (**B**) with two typical height profiles (**C**,**D**) showing from a single chain of 0.5 nm to bundles compose of 2 and 3 chains of [Cd(6-MP$^-$)$_2$·2H_2O].

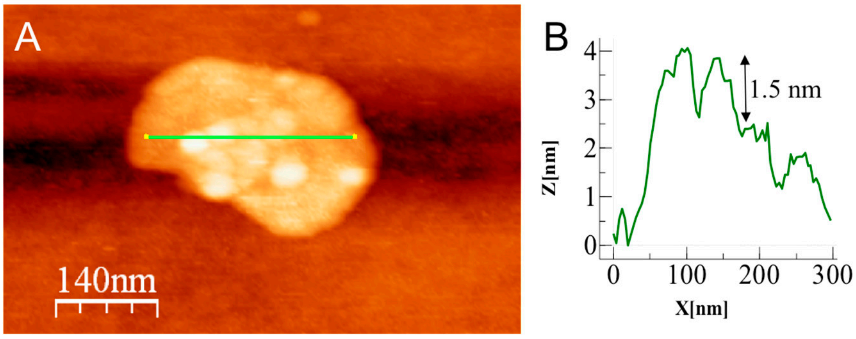

Figure S14. (**A**) AFM topography image of a single sheet of **1D/2D hybrid** nanomaterial drop-casted on SiO_2 substrate, and (**B**) its height profile showing adsorption of *ca*. one to three chains of $[Cd(6\text{-}MP^{-})_2{\cdot}2H_2O]$.

© 2015 by the authors; licensee MDPI, Basel, Switzerland. This article is an open access article distributed under the terms and conditions of the Creative Commons by Attribution (CC-BY) license (http://creativecommons.org/licenses/by/4.0/).
